# Circadian rhythm and its association with birth and infant outcomes: research protocol of a prospective cohort study

**DOI:** 10.1186/s12884-020-2797-2

**Published:** 2020-02-11

**Authors:** Satvinder Kaur, Ai Ni Teoh, Nurul Husna Mohd Shukri, Siti Raihanah Shafie, Normina Ahmad Bustami, Masaki Takahashi, Pei Jean Lim, Shigenobu Shibata

**Affiliations:** 1 Faculty of Applied Sciences, UCSI University, No. 1, Jalan Menara Gading, UCSI Heights 56000 Cheras, Kuala Lumpur, Malaysia; 2 Department of Nutrition and Dietetics, Faculty of Medicine and Health Sciences, Universiti Putra Malaysia, Seri kembangan, Malaysia; 3 School of Healthy Aging, Medical Aesthetics and Regenerative Medicine, Faculty of Medicine and Health Sciences, UCSI University, Kuala Lumpur, Malaysia; 4 Waseda Bioscience Research Institute, Waseda, Singapore; 5 Department of Electrical Engineering and Biosciences, School of Advanced Engineering and Sciences, Waseda University, Tokyo, Japan

**Keywords:** Circadian system, Pregnancy, Chrononutrition, Infant growth, Melatonin, Cortisol

## Abstract

**Background:**

Circadian rhythm plays an important role as our internal body’s clock that synchronizes behavior and physiology according to the external 24-h light-dark cycle. Past studies have associated disrupted circadian rhythm with higher risk of miscarriages, preterm birth and low birth weights. This paper described the protocol of a prospective cohort study which aims to determine the circadian rhythm in pregnant women, identify its association with maternal factors during pregnancy, gestational weight gain, birth and infant outcomes.

**Methods:**

Ten government maternal and child health clinics in Kuala Lumpur, Malaysia will be randomly selected. Sample size of 438 first-trimester pregnant women will be followed-up until the birth of their infant. Salivary melatonin and cortisol concentration among subsample will be determined using enzyme-linked immunosorbent assay. Data on sleep quality, psychological distress and morningness/eveningness chronotype of pregnant women will be collected using validated questionnaires. Pedometer will be used to measure 5-day physical activity data. Total gestational weight gain will be determined at the end of pregnancy. Utilization of 3-day food record is to capture meal timing and nutrient intake. All measurements will be done in 2nd and 3rd trimester. Birth outcomes will be collected through clinic records and Centers for Disease Control and Prevention (CDC) Neonatal questionnaire. Infants will be followed-up at 6 and 12 months old to obtain anthropometric measurements.

**Discussion:**

There is a growing recognition of the role of maternal circadian rhythm, which entrains fetal circadian rhythms that may subsequently have long-term health consequences. The present study will identify the effect of circadian rhythm on pregnancy outcomes and infant growth in the first year of life.

## Background

Circadian rhythm is an endogenous process that has a periodicity of approximately 24 h [1]. It stimulates anticipation of regular and daily repeating events that take place at approximately the same time of day in organism, allowing the upregulation of most of the major physiological systems in mammals [2]. Circadian system in mammals is organized in a hierarchic manner in which the master (central) clock located in the suprachiasmatic nuclei (SCN) of the anterior hypothalamus govern this process [1, 3]. The SCN coordinates other central circadian oscillators including the hypothalamus and pituitary gland, which subsequently organizes the peripheral (local) clocks located in the peripheral tissues and aligns the entire circadian system to the external light/dark cycle [1, 3]. This allows organisms to coordinate endogenous and behavioral activities to the time of day, promoting internal and external synchronization.

Melatonin and cortisol are the most widely used biological phase markers to examine circadian rhythm [4, 5]. The secretions of these hormones are highly rhythmic, with melatonin exhibiting maximal level in the middle of night and gradual decline towards dawn while cortisol peaks in the second half of the night toward early morning, and declines to half of the peak value in the afternoon [6, 7]. Both melatonin and cortisol play a role in synchronizing the circadian rhythms in peripheral tissues to the 24-h pattern and likely provide feedback to the SCN [8].

Many emerging studies addressed the recognition of the importance of maternal circadian rhythm on pregnancy and fetal development. Throughout gestation, the fetus is inevitably exposed to maternal rhythms such as body temperature, food intake and melatonin level [9]. Entrained 24-h rhythms of fetal heart rate, movements and hormones were reported in human, non-human primates and sheep, suggesting that maternal circadian signals may contribute to the entrainment of the peripheral clock in fetus [9]. A study by Seron-ferre and his co-researchers proposed that fetal SCN and fetal organs are peripheral maternal circadian oscillators, which are entrained by maternal signals [9]. This results in internal temporal order during fetal growth, which subsequently allows for postnatal integration of the scattered fetal peripheral circadian clocks into an adult-like circadian system [9].

With the growing evidences that demonstrate the long-term impact of pregnancy environment on the development of fetus and the role of circadian system in governing physiological process and metabolic balance, identifying factors associated with circadian rhythm and its relation to pregnancy and infant growth is imperative. Understanding the circadian factors associated with pregnancy has practical importance for preventive care in ensuring a successful pregnancy and optimal development of the fetus, which is essential to lower the risk of developing postnatal diseases of an individual in later life. Therefore, this study intended to fill current research gaps by investigating the association between maternal circadian rhythm with total gestational weight gain, birth and infant outcomes, as well as determining the lifestyle factors affecting maternal circadian rhythm during pregnancy.

## Methods/design

### Study aims

The aim of the study is to determine the association of stress, physical activity and chrononutrition with circadian rhythm among pregnant women and its impact on total gestational weight gain, birth and infant outcomes. To achieve the aim, the specific research objectives to be investigated in the study are:

1. To determine the circadian rhythm of pregnant women in Kuala Lumpur, Malaysia.
2. To determine the association of stress, physical activity and chrononutrition (energy and nutrient intake, energy and nutrient distribution, meal frequency, eating window, breakfast-skipping, late-night eating and intermittent fasting) with circadian rhythm among pregnant women.
3. To determine the association of irregular circadian rhythm with total gestational weight gain, birth outcomes (birth weight, gestational age at birth and delivery method) and infant outcomes (infant weight, body composition and temperament).

The hypothesis is that mothers with irregular circadian rhythm during pregnancy are associated with higher risk of poor birth outcomes and have unfavorable outcomes, especially on the infant growth and temperament in later life.

### Study design

**Fig. 1:**
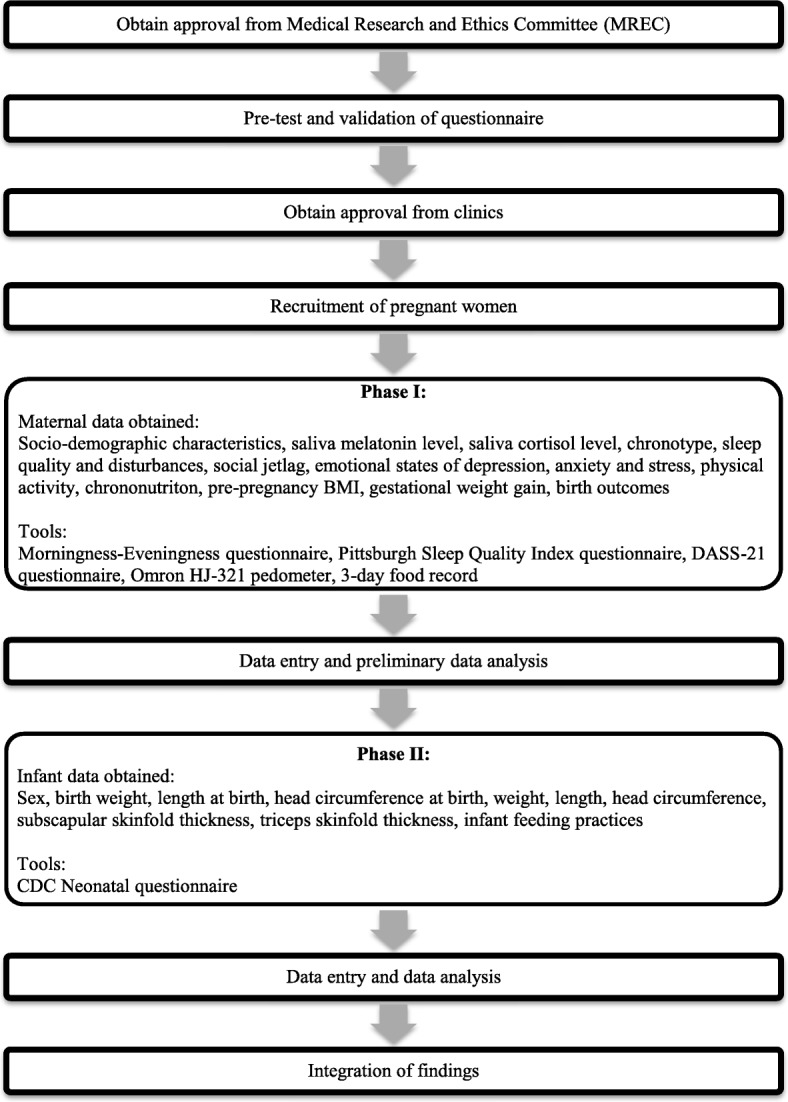
Flowchart of study design

This prospective cohort study will approach pregnant women from their pregnancy at second and third trimester up to delivery and their infants will be followed up at the age of 6 and 12 months old respectively. The whole study is divided into two phases. Phase I (follow-up of pregnant women) will be conducted from 2019 to 2020. Data collection will be conducted during the second (gestational week 13 to 27) and third (gestational week 28 to 40) trimester of pregnancy, and upon delivery of the infant. Circadian rhythm of the pregnant women will be assessed using validated questionnaires based on their sleep quality and disturbances, stress, physical activity and chrononutrition. Pre-pregnancy BMI of the pregnant women will be obtained from all participating clinics record. Gestational weight gain of the pregnant women will be monitored throughout the gestation while information on birth outcome will be collected upon delivery. Phase II (follow-up of infant) will be conducted from 2020 to 2021 whereby infants will be followed-up at 6 and 12 months of age to obtain anthropometric measurements. Fig. 1 depicts the flow diagram of the study.

### Site and participation selection

The study will be carried out in the Federal Territory of Kuala Lumpur, which is the capital city of Malaysia located within the central area of Klang Valley. Klang Valley is made up from different ethnic groups, consisting of Malay (50.6%), Chinese (29.0%), Indian (11.7%), other ethnic group (0.7%) and non-Malaysian citizen (8.0%) [10]. Kuala Lumpur is the most densely populated city with 7366 people per square kilometer despite a population of 1.79 million people. The population is comprised of 58.5% of women of reproductive age ranging from 15 to 49 years [11].

Simple random sampling will be used to select maternal and child clinics to reduce sampling bias. The study will be conducted at 10 randomly selected government Maternal and Child Health clinics (Klinik Kesihatan Ibu dan Anak) in Kuala Lumpur, Malaysia. Randomization will be stratified by clinic to ensure balanced allocation of participants at each of the ten clinics, with further stratification by ethnicity. The Maternal and Child Health clinics are the primary source providing antenatal and postnatal care to pregnant women. First antenatal check-up will be done in the clinic. Subsequent check-ups and appointments will be made once a month until the 28th week, fortnightly from 29th to 36th week and once a week from the 37th week of pregnancy until delivery. Subjects will be follow-up through clinic’s appointment and individual antenatal booklet.

### Subject recruitment

Recruitment of subjects will commence upon obtaining approval from the respective clinics. Purposive sampling method will be used to recruit subjects in their first trimester who fulfil the following inclusion and exclusion criteria, and those who give their consent to participate. Pregnant women who attend the clinics for their routine antenatal care at 1–12 weeks will be approached and invited to participate in this study. The study objectives and procedures will be explained to the subjects and the written consent form will be signed. Information sheet will be provided for their better understanding of the study. Subjects will be asked to sign a written informed consent if they agree to participate in the study on the spot. Additional written consents will be collected from subsample of pregnant women who agree to contribute their saliva samples for circadian melatonin and cortisol rhythm analysis. Sufficient time will be given for subjects to consider their participation in the study and they may contact the research assistant should they agree to participate. Recruitment of subjects began in June 2019 and is currently on-going. To minimize loss to follow-up, efforts will be made to maintain personalised contact with the subjects and incentives will be provided.

### Inclusion and exclusion criteria

Eligible subjects must meet all the following inclusion criteria:

1. Malaysian women aged 19–39 years old.
2. Able to read, write and understand English/Malay language.
3. Singleton pregnancy.
4. Nulliparous.

Subjects meeting any of the following criteria will be excluded from participation in the study:

1. Develop any co-morbidities during the study period.
2. Handicap.
3. Use of recreational drugs/cigarette smoking.
4. Having medication that is known to affect sleep and melatonin or cortisol secretion.
5. Shift workers.
6. Transmeridian travel for the prior 3 months.
7. Pre-existing diabetes mellitus, hypertension and anemia prior to pregnancy.

### Sample size calculation

Sample size was calculated using a formula for cohort study with two proportions. Based on the probability of low birth weight among pregnant women with irregular circadian rhythm (p1 = 0.0388) and probability of low birth weight among pregnant women with regular circadian rhythm (p2 = 0.0313), with 95% power and 5% significance level, a total of 365 of subjects are required for the study [12]. To compensate non-compliance and non-response among subjects including loss of follow up, the sample size is increased (+ 20%) to 438 pregnant women.

### Subsample size calculation

Given the resource constraints, only a subsample will be studied for the analysis of circadian melatonin and cortisol rhythm from saliva samples. Subsample size is calculated using G*Power software, with expected effect size of 0.82, type I error rate of 0.05 and a desired power of 0.95 [13]. The total subsample size is 80 as calculated using G*Power software. To compensate for non-compliance and non-response among pregnant women, the subsample size is increased (+ 20%) to 100.

### Study measurements

Socio-demographic background of subjects, which include their age, ethnicity, educational level, occupation, household income and health history will be obtained through a questionnaire at recruitment. Table 1 shows the details of the variables assessed at each follow-up in this study.

**Table 1:** Summary of data collection and timeline

	Prenatal			Postnatal		
Data	Recruitment	2nd trimester	3rd trimester	Delivery	6 months	12 months
Mothers						
Sociodemographic	•					
Date of birth	•					
Ethnicity	•					
Marital status	•					
Educational level	•					
Occupation	•					
Monthly household income	•					
Health history	•					
Pre-pregnancy BMI	•					
Weight at assessment		•	•			
DASS-21		•	•			
3-day food record		•	•			
Pittsburgh Sleep Quality Index		•	•			
Social jetlag		•	•			
Morningness-Eveningness Questionnaire		•	•			
Salivary sample		•	•			
Step counts		•	•			
Neonatal Questionnaire				•		
Infant						
Sex				•		
Mode of delivery				•		
Birth weight				•		
Length at birth				•		
Head circumference at birth				•		
Infant feeding practice					•	•
Weight					•	•
Length/height					•	•
Head circumference					•	•
Waist circumference					•	•
Mid-upper arm circumference					•	•
Subscapular skinfold thickness					•	•
Triceps skinfold thickness					•	•

### Exposures measures

#### Circadian rhythm

Circadian rhythm of pregnant women will be assessed using both subjective and objective measurements during second and third trimester of pregnancy. Subjective measurement of chronotype (morning and evening types) will be collected using Morningness-Eveningness questionnaire (MEQ) while objective measurement of circadian rhythm will be determined from salivary melatonin and cortisol rhythm of pregnant women. Chronotype refers to the expression of circadian rhythmicity that may vary among individuals [14]. It was demonstrated in past studies that different chronotypes exhibit vary circadian rhythms of several physiological variables [14]. Besides, sleep parameters are useful as phase markers of the circadian rhythm as well, given that sleep/wake cycle is an important aspect of circadian rhythm [5]. In this study, sleep disturbances and quality of pregnant women during second and third trimester will be determined using Pittsburgh Sleep Quality Index (PSQI) Questionnaire.

##### Morningness-Eveningness questionnaire

The Morningness-Eveningness Questionnaire (MEQ) is the most widely used questionnaire to differentiate individuals with extreme circadian tendencies [15]. This questionnaire is frequently used to correlate with core parameters of human circadian organization such as the timing of sleep and predict the endogenous circadian phase [5, 15]. The MEQ score is negatively correlated with the objective phase marker in which subjects with a later circadian phase generally scored lower on the MEQ [15]. The MEQ contains 19 questions, most of which elicit preferences in timing of daily activities and sleep. Most questions in MEQ are designed in a preferential manner where respondent is asked to indicate his/her preferred time of rising and bedtime, as well as physical and mental performance and alertness after rinsing and after different activities [16, 17]. This questionnaire comprises of five behavioral categories: definitive morning (score = 70–86), moderate morning (score = 59–69), neither types (score = 42–58), moderate evening (score = 31–41) and definitive evening (score = 16–30).

##### Salivary melatonin and cortisol rhythm

In the present study, melatonin and cortisol will be measured using saliva samples. Both melatonin and cortisol can be measured in saliva in their free form [7]. Salivary concentrations have been shown to give an accurate indication of free or biologically active melatonin and cortisol as compared to human serum [5, 18]. Measuring salivary melatonin and cortisol can be used to characterize production patterns over each 24-h study period to facilitate comparisons between individual. Moreover, saliva is preferable to serum as a non-invasive alternative to determine melatonin and cortisol level. The taking of blood can cause variation in salivary cortisol level due to the effect of stress [7, 19].

Passive drool method will be used whereby subjects need to tilt their head forward and drooled through a funnel into a centrifuge tube [20]. Saliva sample collection will be taken at 2 time points during pregnancy, at second and third trimester respectively. The first sampling process will start at awakening in all cases, followed by 9:00, 15:00, 21:00 and 03:00 taken at 6 hourly intervals over the 24-h period. This will be done by subjects themselves and each subject will receive individual education regarding the collection process prior to the start of the data collection. To avoid saliva sample contamination with food, subjects are advised to refrain from eating a major meal or brushing their teeth with toothpaste within 30 min of sample collection [21, 22]. No chocolate or bananas, alcohol, caffeine, nicotine or drinks with artificial colorants should have been taken within the prior 12 h and on the day of sampling [21–23].

**Fig. 2:**
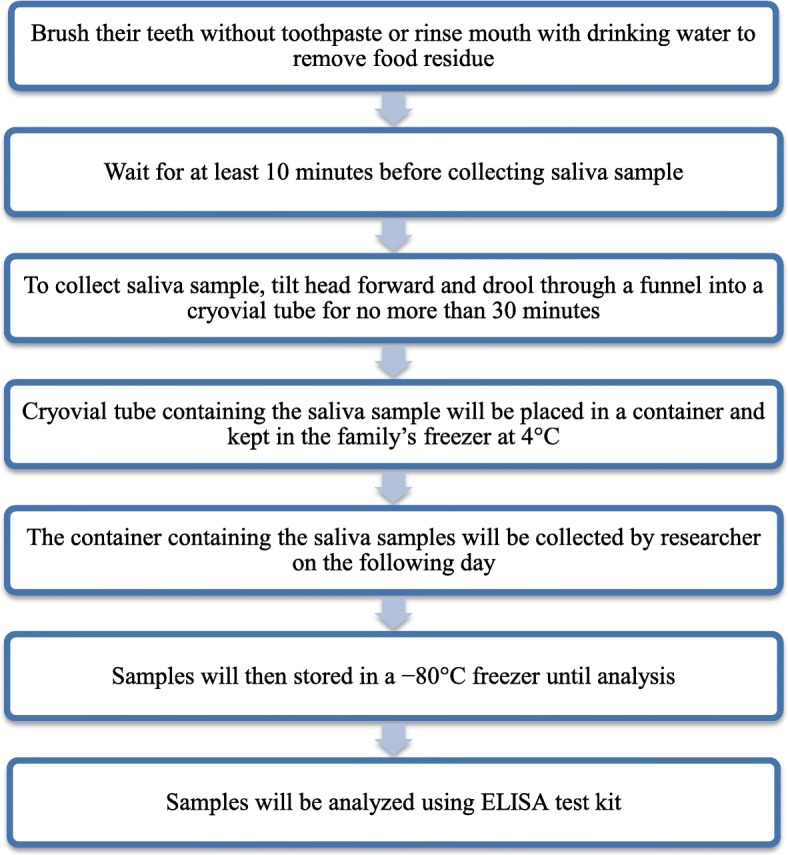
Flowchart of saliva sample collection by passive drool method

During the daytime sampling, saliva samples will be collected under normal light conditions. To minimize light interference with the secretion of salivary hormones, subjects are required to carry out the entire procedure in dim light for collection timed at 03:00 and 21:00 [24]. Subjects will be reminded not to cough up mucus during saliva collection. The centrifuge tube will be covered with aluminium foil and marked with a red line to emphasize the 3 mL marking. After each collection, the tube containing the saliva sample will be placed in a labelled resealable bag in the family’s freezer. Subjects are required to label the tube with the sampling time and date. Subjects will be encouraged to report their actual sampling times, even if there is deviation from the given protocol. Subsequently, the bag with saliva samples will be collected from the subject. The samples will then be centrifuged upon returning to the lab. Supernatant solution will be collected after centrifugation and stored in a − 20 °C freezer until further analysis. Concentration of salivary melatonin and cortisol will be determined using ELISA kit according to manufacturer-provided standards and protocols. Flowchart illustrating the procedures of passive drool method is shown in Fig. 2.

##### Pittsburgh sleep quality index questionnaire

Pittsburgh sleep quality index (PSQI) is a self-rating questionnaire that measures sleep quality and disturbances over a 1-month period [25]. PSQI contains 19 items which summarize 7 clinically derived components of sleep quality and patterns: subjective sleep quality, sleep latency, sleep duration, sleep efficiency, sleep disturbances, use of sleep medication and daytime dysfunction. These items are scored from 0 to 3, and then added together to calculate a total PSQI global score ranging from 0 to 21. It was reported in past studies that the PSQI has similar psychometric properties in both non-pregnant populations and pregnant women [26]. The PSQI has been used on pregnant Asian women in previous studies [26–28]. A validated Malay version of PSQI will be used in the study [29]. The established cutoff of above 5 will be used in this study to depict poor sleep quality in pregnant women. Additionally, bedtime and awake time on workdays and work-free days will be obtained to assess social jetlag (SJL). SJL is calculated as the absolute difference between mid-sleep on work-free days and mid-sleep on workdays [30].

#### Depression, anxiety, and stress scales (DASS-21) questionnaire

The Depression, Anxiety and Stress Scale - 21 Items (DASS-21) is a set of three self-report scales designed to measure the emotional states of depression, anxiety and stress. DASS-21 contains three subscales that cover depression (7 items), anxiety (7 items), and stress (7 items). Each item is scored from 0 (at all) to 3 (very much). Final score of each subscale will be multiplied by two (× 2) as DASS-21 is short form version of DASS which has 42 items. The final scores will be classified into different level of severity: normal, mild, moderate, severe and very severe. A valid Malay version of DASS-21 will be used in current study [16]. DASS-21 has been applied in past studies involving pregnant Asian women, including Malaysian pregnant women [17, 31].

#### Measurement of physical activity

Omron HJ-321 pedometer will be used to measure relative step count in pregnant women. It has been shown to produce accurate measurement of physical activity in the past studies [32–35]. Pedometer is a cost-effective alternative for objective measurement of physical activity and able to capture low-intensity activities including walking [36]. Walking has been reported to be the most popular form of activity for pregnant women in various studies [37–39].

Measurement of relative step count of pregnant women will be taken during second and third trimester. Pedometer readings will be accumulated for a 5 consecutive days during waking hours (excluding water activities), including at least one weekend day, which has previously shown to be sufficient for estimating weekly physical activity [40]. The pedometers will be programmed to an average stride length of 50 cm as used in the previous study [39]. Subjects will be instructed to carry the pedometer around the neck with a lanyard for 5 days to record activity data. They will be given a simple exercise diary to record their daily step count. Pregnant women are encouraged to maintain their normal daily activities to reduce reactivity bias. A full day was considered as wearing the pedometer for at least eight daytime hours and a half day was considered as < 8 h but more than 3 h. If worn for < 3 h a day, this was treated as a missing day. Step count measured by the pedometer will be classified as followed: < 5000/day as “sedentary”, 5000–7499 steps/day as “low active”, 7500–9999 as “somewhat active” and > 10,000 as physically active based on the criteria proposed by [41].

#### 3-day food record

A 3-day food record will be used to record timing of meals taken, type of food or beverage and the amount consumed. Dietary intakes will be assessed for 3 non-consecutive days within a week, comprising two weekdays and one weekend day as the dietary pattern may vary on weekday and weekend day. Subjects will be given instructions on how to record their food intake. Subjects will be encouraged to include detailed descriptions of all foods and beverages including cooking methods, estimated portion size and brand of processed foods and beverages consumed for the past 24 h. Estimation of foods consumed based on household measurement such as cups, plates and tablespoons will be required. To minimize recall bias, subjects will be educated by trained interviewers and they are advised to self-record their diet a the time the food are eaten.

Data that will be identified from the 3-day food record includes eating behaviors including breakfast-skipping, late-night eating and intermittent fasting. Energy and nutrient distribution among the meals will be tabulated. Duration of the last meal of the day to sleeping time will be calculated. Meal frequency as well as eating window will be assessed. Eating window is defined as the time interval between first and last energy-containing meal taken for the day [42].

The estimated amount consumed will then be converted into grams. The mean values for energy and nutrient intake for the three days will be calculated. Malaysian Food Composition Tables [43] and ASEAN Food Composition Tables [44] will be used in dietary analysis. The mean values for energy and nutrient intake will be analyzsed using Nutritionist Pro (First Data Bank Inc., 2011) and then compared with Recommended Nutrient Intake (RNI) for pregnant women [45] to determine intake adequacy. All results will be presented as means, standard deviations and percentages. Ratio between reported total energy intake (EI) and basal metabolic rate (BMR) will be calculated to identify under-reporting of energy intake. BMR is calculated using the BMR equation for Malaysian adults and additional energy requirements during pregnancy will be included in the calculation: 280 kcal/day for second trimester and 470 kcal/day for third trimester [46]. Golderg cut-off point for under-reporting of EI (EI/BMR ratio < 1.2) will be used [47].

#### Pre-pregnancy body mass index (BMI)

Pre-pregnancy BMI will be obtained from clinic records. BMI will be computed using Quetelet’s Index. World Health Organization (WHO) classification of BMI will be used for weight status classification [48]. BMI classification is divided into six categories based on WHO recommendations as depicted in Table 2.

**Table 2:** BMI classification according to WHO guidelines [47]

Classification	BMI range (kg/m^2^)
Underweight	< 18.5
Normal	18.5–24.9
Overweight (Pre-obesity)	25.0–29.9
Obese class I	30.0–34.9
Obese class II	35.0–39.9
Obese class III	≥40

#### Outcomes measures

The main outcomes of this study are birth outcomes and infant outcomes which will be assessed at 6 and 12 months of age, respectively. The secondary outcomes include gestational weight gain (GWG) and infant feeding practices.

#### Gestational weight gain

Range of mean weight gain per week will be determined by deducting weight at assessment with pre-pregnancy maternal weight. Total GWG will be calculated as the differences between maternal weight measured at the last antenatal checkup (at or after 37 weeks period of amenorrhea) and pre-pregnancy weight recorded at the first antenatal appointment at the clinic (measured at < 12 weeks of amenorrhea) [49]. Weight gain recommendation for pregnancy will then be compared with the guidelines established by US Institute of Medicine (IOM). There are three classifications of GEG according to IOM guidelines which are inadequate, adequate and excessive. IOM guidelines on cutoff points for gestational weight gain are illustrated in Table 3.

**Table 3:** Institute of Medicine (IOM) guidelines on gestational weight gain [12]

Pre-pregnancy BMI	Pregnancy weight gain goals (kg)
Underweight	12.5–18
Normal	11.5–16
Overweight	7–11.5
Obese	5–9

#### Birth outcomes

Birth outcomes such as birth weight, gestational age at birth, delivery method, duration of labor, instrument-assisted labor and other information about the experience during labor will be collected from clinic record. Data on low birth weight, preterm birth and intrauterine growth restriction (IUGR) will be identified through clinic records. Preterm birth is defined as a gestational age less than 37 weeks which may contribute to low birth weight with birth weight less than 2500 g [50]. IUGR refers to a condition in which the rate of fetal growth is below normal in light of the growth potential of a specific infant as per the race and gender of the fetus [51]. An infant with IUGR is defined as being below the 10% percentile of the recommended gender-specific birthweight for gestational age according to William’s curve [52, 53]. Focus on the birth outcome will be limited to the 3 major adverse birth outcomes: low birth weight, preterm birth, and intrauterine growth restriction (IUGR). These adverse birth outcomes are the leading causes of perinatal morbidity, mortality, neurodevelopmental impairments and disabilities among newborn babies [53].

#### CDC neonatal questionnaire

Neonatal questionnaire from Infant Feeding Practices Study (IFPS) II will be used to assess factors that affect infant feeding choices [54]. Questions on early feeding practices, including breastfeeding status, sources of information and sources of support are included in the questionnaire. These information are crucial as early feeding choices are crucial in influencing infant growth, especially in the first year of life.

#### Infant outcomes

##### Infant anthropometric assessment

Data on infant anthropometry will be assessed from infant book record at 6 and 12 months of age respectively. Anthropometry data of infants which comprises of head circumference, length and weight will be collected. Data will then be plot using WHO Growth Charts (include the reference, whereby weight for age, head circumference for age, length for age will be used).

##### Revised infant behavior questionnaire (IBQ-R)

Infant temperament refers to behavioral tendencies that is observed early in life and stable across age [55]. It is an important aspect that might contribute to infant physical growth [55]. Thus, infant temperament will be measured at 14–16 weeks using the validated RIBQ based on a 7-point Likert scale, from 1 (never) to 7 (always). Three major dimensions will be assessed: surgency/extraversion, negative affectivity and orienting/regulation. The IBQ-R has been validated for use for infants aged 3 to 12 months old [56] and its reliability has been reported in previous studies [57].

### Statistical analysis

All statistical analysis will be performed using SPSS software version 20 (SPSS Inc., Chicago, IL, USA). Continuous variables are presented as mean ± SD. Statistical probability level of *p* < 0.05 will be considered significant. Normality of continuous data will be tested using Q-Q Plot, histogram and Kolmogorov-Smirnov Test. Data of chronotype, sleep quality, depression, anxiety and stress, physical activity, BMI and GWG will be described in qualitative form. We will perform statistical analysis of the data quantitatively to examine the association between variables.

To determine circadian rhythm, we will perform circadian analysis of salivary melatonin and cortisol levels using Acrophase software. Circadian pattern of melatonin and cortisol secretion in 24-h will be represented in a graph. Circadian variables such as baseline (daytime melatonin level), acrophase (clock time at which the melatonin reaches peak level) and amplitude (difference between baseline and peak level) will be extracted from circadian analysis. Diurnal cortisol slope will be tabulated based on two data points (awakening and 21:00) by subtracting value at 21:00 from awakening value, and dividing by the number of hours between these two time points. Chronotype of respondents will be presented as categorical variables of Morning-type (M-type), Neither-type (N-type) and Evening-type (E-type). Student’s paired t-test will be used for comparing circadian variables during second and third trimester. Correlation between chronotypes and sleep quality will be assessed using Pearson’s correlation test.

We will perform regression to determine the association between circadian variables and depression, anxiety and stress scores and step count. Energy and nutrient intake as well as distribution among meals. Multivariable generalised linear models will be used to examine the associations between chrononutrition data (energy and nutrition intake as well as distribution between meals, duration between the last meal and sleeping time, meal frequency and eating window). We will also perform ANOVA to analyse the association between circadian variables with eating behaviors including breakfast-skipping, late-night eating and intermittent fasting.

To further examine the association between the circadian variables with total gestational weight gain, birth outcomes (birth weight, gestational age at birth), infant outcomes (weight, body composition and infant temperament scores), multiple linear regression will be used, with covariates such as maternal age and pre-pregnancy BMI. Odds ratios and 95% confidence intervals will be used to determine the strength and precision of each association after adjusting for potential confounders.

## Discussion

Pregnancy is a critical period when the system and organs of a new human body develops. Any disruption to the environment in which the fetus grows may result in developmental adaptations that produce permanent alterations to structural and physiological metabolic functions of the fetus [58, 59]. A hypothesis proposed in 1990 by the British epidemiologist David Barker, known as the Barker hypothesis, states that the environment during embryonic and fetal development increases the susceptibility of developing certain postnatal diseases later in life [60]. These effects are referred to as fetal programming which highlights the potential link between maternal factors on fetal growth and subsequently, infant health.

Pregnancy is considered as a period susceptible to chronodisruption as pregnant women often undergo physical, physiological, social and emotional changes, which may alter their usual lifestyle. Sleep problems such as insomnia and sleep deficiency are among the common issues reported during pregnancy due to various factors such as endocrine changes, backache, increase in size, fetal movement, frequent urination, leg cramps, abdominal discomfort, heartburn and vomiting [61, 62]. Sleep/wake cycles are closely intertwined with the circadian system, and disrupted sleep during pregnancy could potentially affect maternal circadian rhythm [5]. Furthermore, due to the lifestyle modernization and common use of artificial lightning, problem of disrupted or irregular circadian rhythm is not limited to shift worker, but may pose a significant health risk on the general public as well.

The strengths of the study include the utilisation of objective and subjective measurement to determine individual aspects of circadian rhythm, namely Morningness-Eveningness questionnaire and salivary melatonin and cortisol rhythms. Measurement of melatonin and cortisol levels at specific time points across the day allows the characterization of the full 24-h rhythm, which gives a whole picture of circadian rhythm. The use of pedometer to measure physical activity could minimize under and over-reporting of physical activity which are common when reported using self-assessment questionnaires. Other than using infant weight and body measurements to assess infant outcomes, this study also looks at infant temperament, which is an important aspect that might contribute to infant physical growth. Data on infant feeding practices will be included as early nutrition plays a crucial role in influencing infant growth.

In term of limitations, multiple assessments during second and third trimester of pregnancy could reduce compliance and elevate maternal stress which in turn can result in increased drop-out rates. However, this could be minimized through frequent and positive interactions to encourage continued participation, such as knowledge sharing session with the subjects at the clinic. Another limitations of study is the lack of control for compliance to the protocol of saliva sampling, e.g. by an electric device. However, several steps are undertaken to ensure compliance, including individual education prior to sampling, use of use-friendly sampling kit with easily understood instructions and appointment will be planned to collect the samples from subjects’ house, which ease the samples returning process. Besides, this study will include parent report of infant temperament and feeding practices which may cause response bias.

Maternal circadian rhythm and its influence on pregnancy and birth outcomes in human studies are scarce. Majority of the past studies on circadian rhythm focuses on shift work and related abnormalities. Hence, there is a need to explore circadian rhythm among the general pregnant women. Data from this study will contribute to the gap in knowledge related to the role of circadian rhythm during pregnancy on birth outcome and infant growth in the first year of life. This study is expected to demonstrate an association between circadian rhythm with total gestational weight gain, birth and infant outcomes. The findings from this study can be used to develop evidence-based recommendations on improving maternal circadian rhythm during pregnancy. By identifying the role circadian rhythm has to play during pregnancy, it could contribute to the development of relevant intervention strategies as well as strengthening existing strategies and policies related to maternal and infant health in Malaysia.

## Data Availability

Not applicable

## Abbreviations

ANCOVA: Analysis of covariance
BMI: Body Mass Index
BMR: Basal metabolic rate
CDC: Centre of Disease Control
DASS-21: Depression, Anxiety and Stress Scale - 21 Items
EI: Energy intake
ELISA: Enzyme-linked immunosorbent assay
GWG: Gestational weight gain
HKL: General Hospital Kuala Lumpur
IFPS: Infant Feeding Practices Study
IOM: Institute of Medicine
IUGR: Intrauterine growth restriction
MEQ: Morningness-eveningness questionnaire
NPANM: National Plan of Action For Nutrition of Malaysia
PSQI: Pittsburgh sleep quality index
RNI: Recommended nutrient intake
SCN: Suprachiasmatic nuclei
SD: Standard deviation
SJL: Social jetlag
WHO: World Health Organization
